# iStent as a Solo Procedure for Glaucoma Patients: A Systematic Review and Meta-Analysis

**DOI:** 10.1371/journal.pone.0128146

**Published:** 2015-05-27

**Authors:** Monali S. Malvankar-Mehta, Yufeng Nancy Chen, Yiannis Iordanous, Wan Wendy Wang, John Costella, Cindy M. L. Hutnik

**Affiliations:** 1 Department of Ophthalmology, Schulich School of Medicine and Dentistry, Western University, London, ON, Canada; 2 Department of Epidemiology and Biostatistics, Schulich School of Medicine and Dentistry, Western University, London, ON, Canada; 3 Schulich School of Medicine and Dentistry, Western University, London, ON, Canada; 4 Allyn & Betty Taylor Library, Natural Sciences Centre, Western University, London, ON, Canada; Harvard Medical School, UNITED STATES

## Abstract

**Background:**

Glaucoma is a leading cause of irreversible blindness. It is firmly entrenched in the traditional treatment paradigm to start with pharmacotherapy. However, pharmacotherapy is not benign and has been well documented to have a number of significant challenges. Minimally invasive glaucoma surgery (MIGS) that targets the outflow pathway with minimal to no scleral dissection has resulted in the need to reconsider the glaucoma treatment paradigm.

**Purpose:**

To perform a systematic review and meta-analysis to evaluate and quantify the effect on post-operative intraocular pressure (IOP) and number of topical glaucoma medications, in patients receiving the iStent MIGS device as the solo procedure without concurrent cataract surgery.

**Methods:**

A systematic review was conducted by searching various databases between January 1, 2000, and June 30, 2014. Studies reporting up to a maximum follow-up period of 24 months were retrieved and screened using the EPPI-Reviewer 4 gateway. Percentage reduction in IOP (IOPR%), and mean reduction in topical glaucoma medications after surgery were computed. Meta-analysis was performed using STATA v. 13.0. The standardized mean difference (SMD) was calculated as the effect size for continuous scale outcomes. Heterogeneity was determined using the *I* ^2^ statistics, *Z*-value, and χ^2^ statistics. Fixed-effect and random-effect models were developed based on heterogeneity. Sub-group analysis was performed based on the number of iStents implanted and the follow-up period. The outcome measures were changes in the IOP and number of glaucoma medications.

**Results:**

The search strategy identified 105 records from published literature and 9 records from the grey literature. Five studies with 248 subjects were included for quantitative synthesis. A 22% IOP reduction (IOPR%) from baseline occurred at 18-months after one iStent implant, 30% at 6-months after two iStents implantations, and 40% at 6-months after implantation of three iStents. A mean reduction of 1.2 bottles per patient of topical glaucoma medications occurred at 18-months after one iStent implant, 1.45 bottles per patient at 6-months after two iStents, and one bottle of medication per patient was reduced at 6-months following placement of three iStents implants. Meta-analysis results showed a significant reduction in the IOP after one iStent (SMD = -1.68, 95% CI: [-2.7, -0.61]), two iStents (SMD = -1.88, 95% CI: [-2.2, -1.56]), and three iStents (SMD = -2, 95% CI: [-2.62, -1.38]) implantation. Results showed a significant drop in the topical glaucoma medications after one iStent (SMD = -2.11, CI: [-3.95, -0.27]), two iStent (SMD = -1.88, CI: [-2.20, -1.56]), and three iStents (SMD = -2.00, CI: [-2.62, -1.38]) implantation. The maximum reduction in IOP occurred at 12-months (SMD = -2.21, CI: [-2.53, -1.88]) and a significant reduction in post-operative topical glaucoma medications occurred even after 18-months of iStent implantation (SMD = -0.71, CI: [-1.15, -0.26]).

**Conclusion:**

iStent implantation as a solo procedure without concurrent cataract extraction does lower IOP, and reduces the dependency on glaucoma medications. This effect seems to last at least 18 months.

## Introduction

Modern medicine has contributed to increased longevity. As the number of older adults explodes globally, implications will be experienced in all facets of life. In 2012, the over-60 population numbered 810 million, quadruple the size of this cohort in 1950, but not even half of the two billion population expected to be over-60 by 2050 [1]. As age is a major risk factor for the most common causes of visual loss, the number of adults with visual disabilities is anticipated to increase substantially.

The fear of vision loss is ranked third after cancer and heart disease [2]. Currently it is estimated that 285 million people worldwide are visually disabled with 39 million [3] being totally blind. Visual impairment is a leading cause of age-related functional disability. In the United States, 3.6 million [4] and in Canada, 1 million [5] people suffer from severe visual impairment to complete blindness. By the age of 65, one in nine Canadians is known to experience irreversible vision loss and by the age of 75, one in four [5] are affected.

Glaucoma is a leading cause of irreversible blindness [6], affecting an estimated 60.5 million people worldwide [2,7], 3 million in the U.S. [8,9], and 400,000 in Canada [6]. It has been estimated that in 2020, primary open-angle glaucoma (POAG), the most common form of glaucoma, will globally plague 80 million people [6,10]. The economic cost of POAG is large with the direct annual cost estimated at 2.86 billion in the U.S. [11] and 300 million in Canada [5].

Lowering the IOP is currently the only treatment known to delay the progression of glaucoma. In the traditional treatment paradigm, pharmacotherapy is the first line of treatment [12]. However, pharmacotherapy is not benign and has been well documented to have a number of significant challenges. Patient non-compliance [13–16], difficulty with administration [17,18], local irritation, and ocular surface toxicity [19–21] have been reported as causative of treatment failure. Systemic side effects have also been reported with topical administration [22,23].

Minimally invasive glaucoma surgery (MIGS) refers to a group of emerging procedures that manipulate the trabecular outflow pathways. According to Food and Drug Administration (FDA), a MIGS device is a type of IOP lowering device used to lower IOP using an outflow mechanism with either an *ab interno* or *ab externo* approach, associated with little or no scleral dissection and minimal or no conjunctival manipulation [24]. The iStent trabecular micro-bypass stent (Glaukos Corporation, Laguna Hills, CA) is an example of one of these MIGS devices [25,26].

The iStent is an implantable device made of titanium and coated with heparin that is inserted *ab interno* into Schlemm’s canal [27]. It lowers IOP by providing a direct channel for aqueous outflow from the anterior chamber to collector channels [28,29]. It has garnered much attention as it is one of the first MIGS devices to achieve relatively widespread use with promising initial reports regarding efficacy and safety. [30–32]. Data also support its potential use as an adjunctive therapy at the time of cataract surgery for patients using multiple glaucoma medications [26,31,33–35]. A cost effective analysis has demonstrated a potentially lower cumulative cost for iStent implantation versus glaucoma medications over time in Canada [36].

Most studies examining the effect of iStent on patients with POAG have been small, non-randomized, and often lacking appropriate control arms. These studies have a considerable variability in follow-up periods, measured outcomes, and definitions of surgical success. The purpose of the current systematic review is to analyze all available data on the iStent as a solo procedure without concurrent cataract surgery as well as to summarize and quantify the effect it has on both intraocular pressure and use of topical glaucoma medications, for patients with POAG.

## Methods

### Literature search strategy

**Fig 1:**
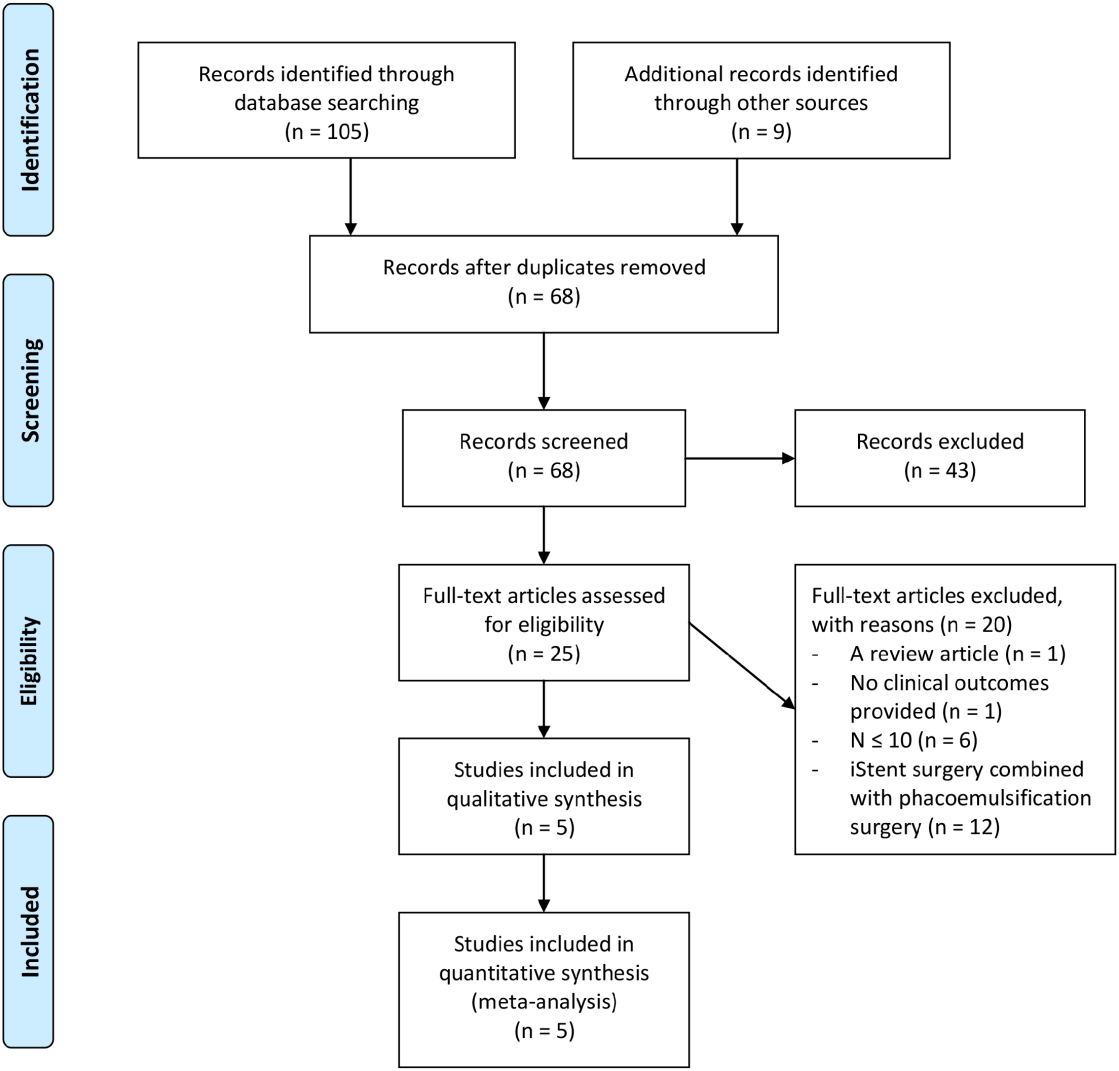
PRISMA Flow Diagram for iStent as a Solo Procedure for Patients with Glaucoma.

The search strategy was implemented using the following databases: MEDLINE In-Process & Other Non-Indexed Citations, MEDLINE Daily and MEDLINE (Ovid), EMBASE (Ovid), BIOSIS Previews (Thomson-Reuters), CINAHL (EBSCO), PubMed, Health Economic Evaluations Database (HEED), Web of Science (Thomson-Reuters) and the Cochrane Library (Wiley). OVID AutoAlerts for MEDLINE and EMBASE were set up to send monthly updates with regard to any relevant new literature. Monthly updates were also performed on HEED, PubMed and Cochrane Library databases. We included any relevant articles published between January 1, 2000 and June 30, 2014. Search strategies were constructed, with the assistance of an information specialist, utilizing database specific subject headings and keywords for “iStent”, and “glaucoma”. Each strategy was modified to complement the specific database and platform. The search strategies for MEDLINE and EMBASE are included in the supplementary material (S2 File). In this research, we adhered to the Preferred Items for Systematic Reviews and Meta-Analyses (PRISMA) guidelines (Fig 1 and S1 File) [37].

Grey literature between January 1, 2000 and June 30, 2014 was identified by searching ProQuest Dissertations and Theses, the Canadian Health Research Collection (Ebrary), as well as any available meeting abstracts of the European Society of Ophthalmology (ESO), Canadian Ophthalmology Society (COS), Association for Research in Vision and Ophthalmology (ARVO), and American Academy of Ophthalmology (AAO). The Conference Proceedings Citation Index was also included as part of the Web of Science search. Google and other Internet search engines were also used to search for additional web-based materials and information. These searches were supplemented by hand searching the bibliographies of all the included studies, and through contacts with appropriate experts and agencies.

### Inclusion and exclusion criteria

Economic studies, comparative studies, observational studies, cohort studies, case series, randomized control trials, clinical trials, multi-center studies, meta-analyses, and systematic reviews were included. Review articles, editorials, opinions, pilot studies, surveys, and case reports were excluded. Studies on human subjects, on adults 18 years and older with mild to moderate glaucoma, and sample size of 20 or more were included. Studies considering complications or explicitly discussing post-operative outcomes were included. Only studies published in English language were included. There was no restriction placed on country in which the study was done or the follow-up period.

All records identified through the literature search process were imported to EPPI Reviewer 4.0 (by EPPI-Centre, Social Science Research Unit, the Institute of Education, the University of London, UK). Duplicates were then removed within EPPI Reviewer and the remaining records proceeded on to the screening stages. The screening process involved 3 levels. Titles of the included records were screened in Level 1, abstracts were screened in Level 2, and full text of the included records was screened in Level 3 (S3 File). Two reviewers independently screened the studies at each level. At the end of each level of screening, agreements and disagreements were calculated using Cohen’s kappa (κ) coefficient. Disagreements were resolved by consensus and if consensus was not reached then a third reviewer intervened to resolve the disagreements.

### Quality assessment

All included articles were scored for quality using the Downs and Black checklist [38]. Total five articles were included, one article was determined to be of high quality [39], one of medium quality [40] while three were considered of poor quality [30,32,41]. A quality check was performed to ensure completeness of our methodology. Due to limited evidence none of the lesser quality articles were excluded from the analysis.

### Data extraction

The following data were abstracted from each article: author name, year of publication, study design, study location, number of iStents implanted, follow-up in months, sample size, patients demographic characteristics, baseline intraocular pressure (IOP), number of pre-operative glaucoma medications, pre-operative best corrected visual acuity (BCVA), other baseline characteristics such as cup to disc ratio, corneal thickness, visual disability score, vision quality of life (QOL) score, and post-operative characteristics including IOP, total number of topical glaucoma medications, compliance to treatment, percentage of patients with 20/40 or better visual acuity, percentage of patients off-medication, percentage of patients with IOP ≤ 18 mmHg, list of reported complications and rates of each complication. Data were extracted by one reviewer, while a second reviewer examined all extracted data. A third reviewer intervened if consensus was not reached.

### Statistical analysis

Meta-analysis was completed using STATA v. 13.0 (STATA Corporation, College Station, TX). The main outcomes of interest were the mean and standard deviation (SD) of pre- and post-operative IOP as well as total number of topical glaucoma medications. In studies, where SD was not reported, data about the range, confidence interval, standard error, and p-value was obtained and then converted to SD value. The extracted mean and standard error of the IOP at baseline and end point were used to compute the mean IOP reduction (*IOPR*), percentage of IOP reduction (*IOPR*%), within group standard error (SE_*IOPR*_), and standard error of percentage of IOP reduction (SE_*IOPR%*_) using the equations below [42]:

$$IOPR=IOP_{baseline}-IOP_{endpoint}$$

$$IOPR\%\;=\;\frac{IOPR}{{IOP}_{baseline}}$$

$${SE}_{IOPR}\;=\;\sqrt{{SE}_{baseline}^{2}+{SE}_{endpoint}^{2}}$$

$${SE}_{IOPR\%}\;=\;\frac{{SE}_{IOPR}}{{IOP}_{baseline}}$$

Standard deviation percentage of IOP reduction (*SD*_*IOPR%*_) was then calculated by the formula ${SD}_{IOPR\%}\;=\;{SE}_{IOPR\%}\times \sqrt{n}$ (Table 1).

**Table 1:** Reported pre- and post-operative intraocular pressure in studies included in meta-analysis.

Author (Year)	Study Design	Study Location	Number of iStent	N	Follow-up (months)	Mean Age	Age (SD)	Baseline IOP (Mean)	Baseline IOP (SD)	Outcome IOP (Mean)	Outcome IOP (SD)	IOPR%	SD_IOPR%
Ahmed (2012) ^[^ ^40^ ^]^	Case Series	Canada	1	30	6	-	-	23.3	1.3	16.1	4.4	31	12
	Case Series	Canada	2	30	6	-	-	23.2	0.9	13.7	2.5	41	11
	Case Series	Canada	3	30	6	-	-	23.2	2.4	13.6	2.1	41	14
Buznego (2009)^[^ ^32^ ^]^	Cohort	U.S.	1	41	18	-	-	21.5	6.9	16.7	6.9	22	45
Charters (2013)^[^ ^30^ ^]^	RCT	U.S.	2	39	-	-	-	30	4.0	13.2	0.0	56	13
Singh (2012)^[^ ^41^ ^]^	Case Series	Canada	2	39	6	73	0	19.1	3.8	15.1	2.7	21	24
	Case Series	Canada	2	39	12	73	0	19.1	3.8	15.9	0.9	17	20
Voskanyan (2014)^[^ ^39^ ^]^	Case Series	France, Germany, Italy, Republic of Armenia, Spain	1	99	1	66.4	10.9	26.3	3.5	17	6.4	41	18
	Case Series	France, Germany, Italy, Republic of Armenia, Spain	1	99	3	66.4	10.9	26.3	3.5	16.6	4.5	41	18
	Case Series	France, Germany, Italy, Republic of Armenia, Spain	1	99	6	66.4	10.9	26.3	3.5	16.8	4.1	41	18
	Case Series	France, Germany, Italy, Republic of Armenia, Spain	1	99	7	66.4	10.9	26.3	3.5	15.8	3.2	41	18
	Case Series	France, Germany, Italy, Republic of Armenia, Spain	1	99	9	66.4	10.9	26.3	3.5	15.5	3	41	18
	Case Series	France, Germany, Italy, Republic of Armenia, Spain	1	99	12	66.4	10.9	26.3	3.5	15.7	3.7	41	18

Weighted mean reduction in IOPR% following two iStents implantation at 6-months: 30.

For continuous scale outcomes such as mean values, standardized mean difference (SMD) was calculated as the treatment effect or effect size. SMD was chosen as the treatment effect since it is a mean difference standardized across all studies. To compute SMD for each study, the difference between the mean pre- and post-operative values for each outcome measure (i.e. IOP, number of glaucoma medications) was divided by the SD for that same outcome measure. Weights were assigned to each SMD according to the inverse of its variance and then average was computed. SMD for each study was then aggregated using the fixed or random-effect model based on the presence of heterogeneity to estimate the summary effect.

To test heterogeneity, *I* ^2^statistics, *Z*-value, and χ^2^ statistics were computed. An *I* ^2^ value of less than 50% implies low heterogeneity, and in these cases, a fixed-effect model was computed. An *I* ^2^statistics of 50% or more represents high heterogeneity, and in these cases a random-effect model was calculated. Additionally, a high *Z*-value, a low p-value (< 0.01) and a large *χ* ^2^ value implies significant heterogeneity and therefore, a random-effect model using DerSimonian and Laird methods was computed. Forest plots were also generated for each case. Funnel plots were generated to check publication bias.

Sub-group analysis was performed to ascertain the influence of the number of iStents implanted and the follow-up period on post-operative IOP and number of topical glaucoma medications. Causes of heterogeneity were also explored.

## Results

### Search results

The search strategy identified 105 records, including 19 from MEDLINE, 29 from EMBASE, 23 from CINAHL, 25 from ISI Web of Science, and 9 from Cochrane Library databases. An additional 9 records were found by grey literature search. Sixty-eight articles remained after removal of duplicates. After screening, a total of 25 records remained. After manually reviewing the 25 full-text articles, five studies (248 subjects) met our inclusion/exclusion criteria and were included for qualitative and quantitative synthesis. Fig 1 shows the PRISMA Flow diagram, outline the screening process.

### Study characteristics

Study characteristics and results are summarized in Tables 1 and 2. Table 1 lists the characteristics of the included studies, as well as the following main outcomes: pre-operative versus post-operative intraocular pressure (IOP), percentage of IOP reduction (*IOPR%*), and standard error of percentage of IOP reduction (*SE*_*IOPR%*_). Table 2 lists pre-operative versus post-operative number of glaucoma medications and mean reduction in medications.

**Table 2:** Reported pre- and post-operative topical glaucoma medications in studies included in meta-analysis.

Author (Year)	Number of iStent	N	Follow-up (months)	Baseline Medications(Mean)	Baseline Medications (SD)	Outcome Medications (Mean)	Outcome Medications (SD)	Mean Reduction in Total Medications
Ahmed (2012) ^[^ ^40^ ^]^	1	30	6	2.0	0.5	1.0	0.5	1.0
	2	30	6	2.0	0.5	1.0	0.5	1.0
	3	30	6	2.0	0.5	1.0	0.5	1.0
Buznego (2009)^[^ ^32^ ^]^	1	41	18	1.6	1.7	0.4	1.7	1.2
Charters (2013)^[^ ^30^ ^]^	2	39	-	1.0	-	0.1	-	0.9
Singh (2012)^[^ ^41^ ^]^	2	39	6	2.5	1.1	0.7	1	1.8
	2	39	12	2.5	1.1	0.52	0.9	1.98
Voskanyan (2014)^[^ ^39^ ^]^	1	99	1	2.21	0.44	-	-	-
	1	99	3	2.21	0.44	-	-	-
	1	99	6	2.21	0.44	-	-	-
	1	99	7	2.21	0.44	-	-	-
	1	99	9	2.21	0.44	-	-	-
	1	99	12	2.21	0.44	0.414	1.0	1.796

Weighted mean reduction in medications following two iStents implantation at 6-months: 1.45.

The number of iStents inserted varied. One study [40] compared the impact of implanting one versus two versus three iStents on post-operative IOP and number of glaucoma medications. Two studies examined the insertion of two iStents. Two studies investigated the impact of one iStent on post-operative IOP and the number of medications. The follow-up periods for each study also varied significantly. One study reported six month follow-up period [40]. Two studies reported data up to 12-months [39,41] and one study reported an 18-months follow-up period [32].

Table 3 lists data on post-operative characteristics of included studies such as complications, complication rates, percentage of patients with good vision, reduced number of medications, and controlled IOP.

**Table 3:** Reported post-operative characteristics and complications of studies included in meta-analysis.

Author (Year)	Post-operative Characteristics				
	Patients with 20/40 or better VA (%)	Patients off-medications (%)	Patients with IOP ≤ 18 mmHg (%)	Patients with IOP ≤ 18 mmHg without medications (%)	List of complications reported(rate of complication in %)
Ahmed (2012)^[^ ^40^ ^]^	-	0	100	-	hypotony (3), hyphema (3)
Buznego (2009)^[^ ^32^ ^]^	-	Most patients were off medication	100	Most patients were off medication	iStent malposition (14.63), iStent replacement (4.87), iStent reposition (2.44)
Charters (2013)^[^ ^30^ ^]^	74	92	92	92	No complications reported
Singh (2012)^[^ ^41^ ^]^	-	0	100	-	No complications reported
Voskanyan (2014) ^[^ ^39^ ^]^	86	66	81	66	Elevated IOP (10.1), iStent obstruction (3), iStent malposition (1), Intraocular inflammation (1), Sub-conjunctival hemorrhage (1), iStent not visible upon gonioscopy (13.1), Goniosynechiae (1), Iris synechiae (1), iStent malposition (1), Allergic reaction to medications (1), Posterior capsular opacification (2)

A blank indicates that information has not been provided in that study.

### Publication bias

**Fig 2:**
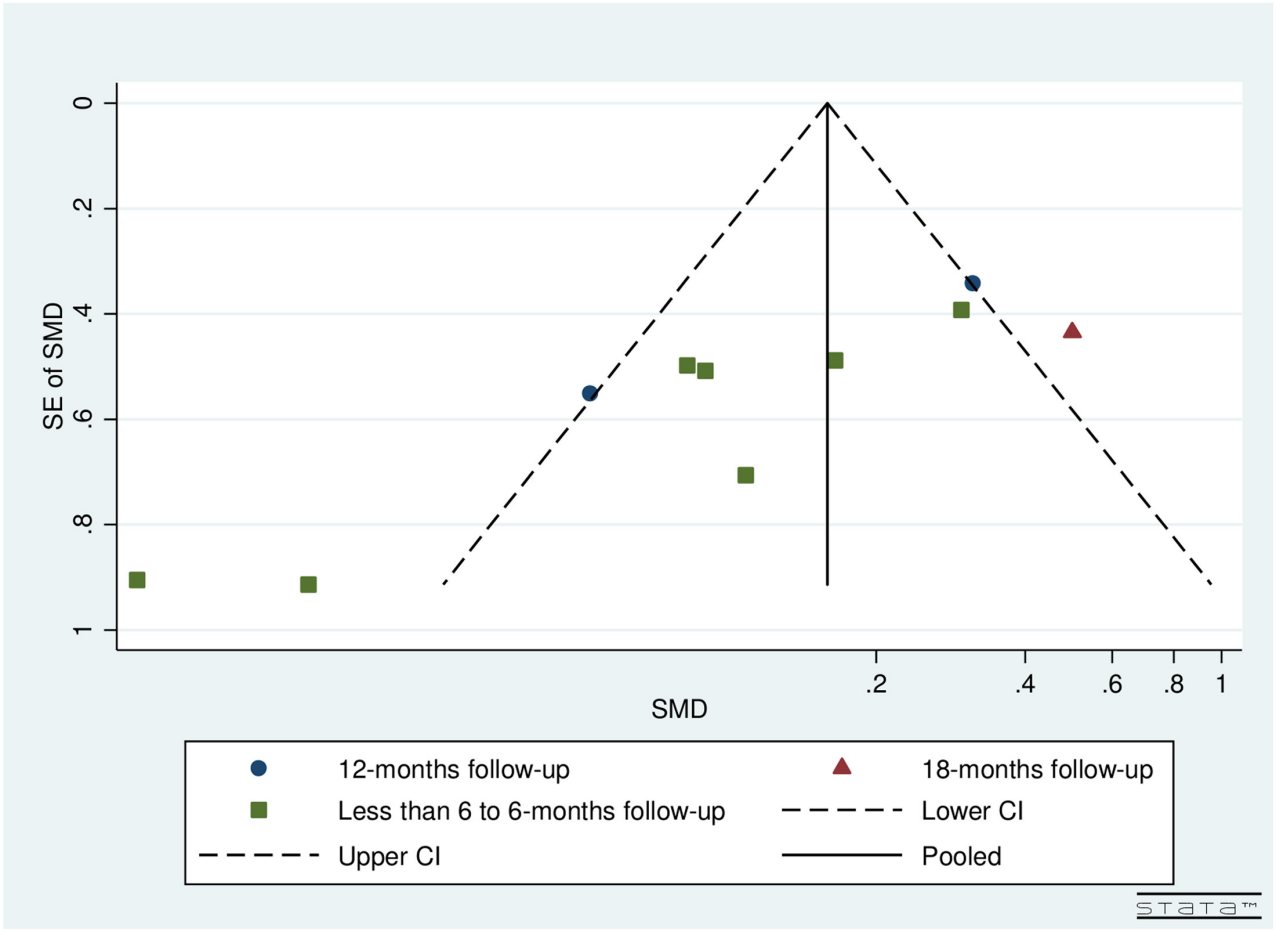
Funnel plot for studies examining pre- and post-operative intraocular pressure (IOP) by follow-up (months).

**Fig 3:**
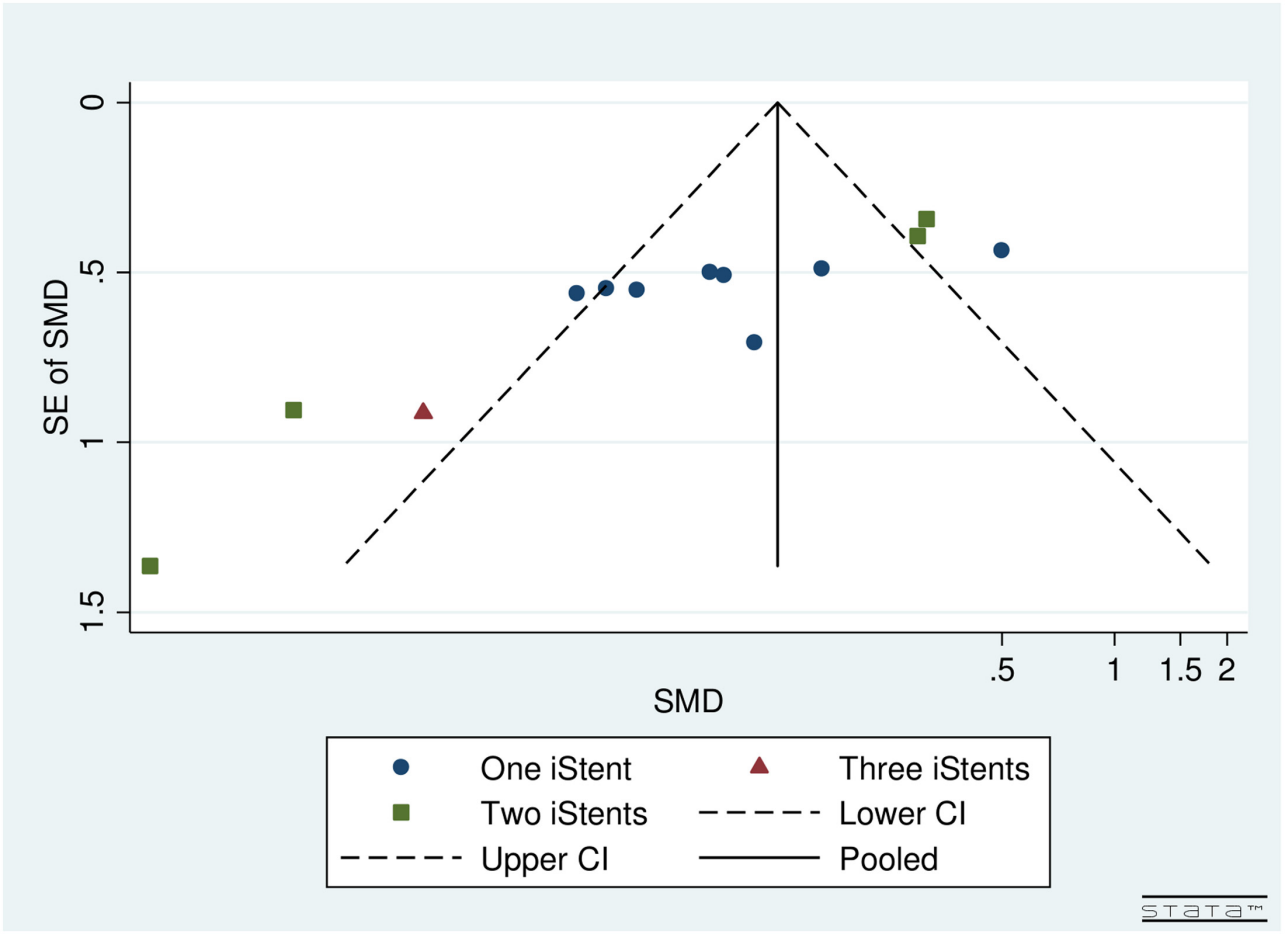
Funnel plot for studies examining pre- and post-operative IOP by number of iStents inserted.

**Fig 4:**
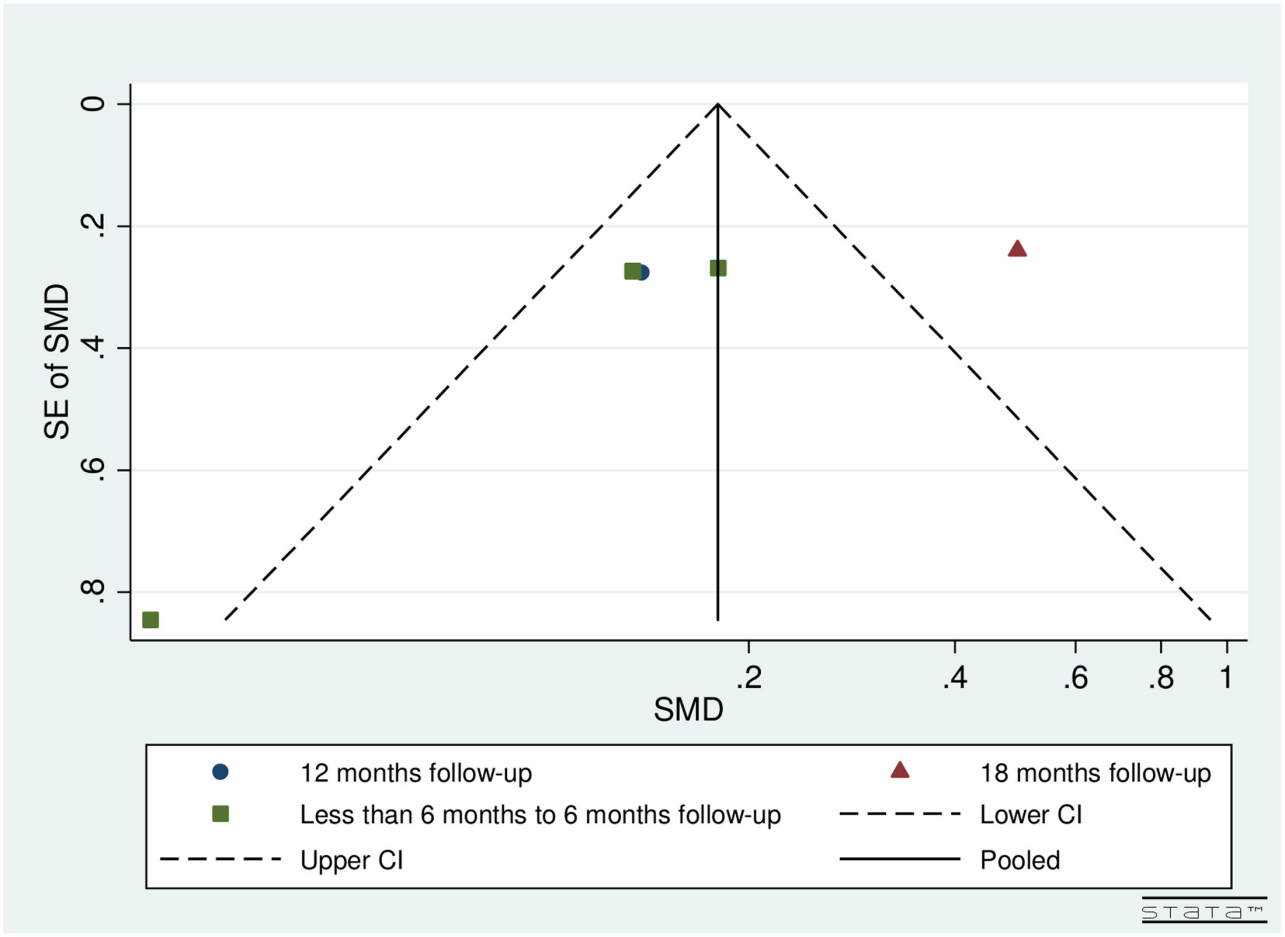
Funnel plot for studies examining pre- and post-operative number of medications by follow-up (months).

**Fig 5:**
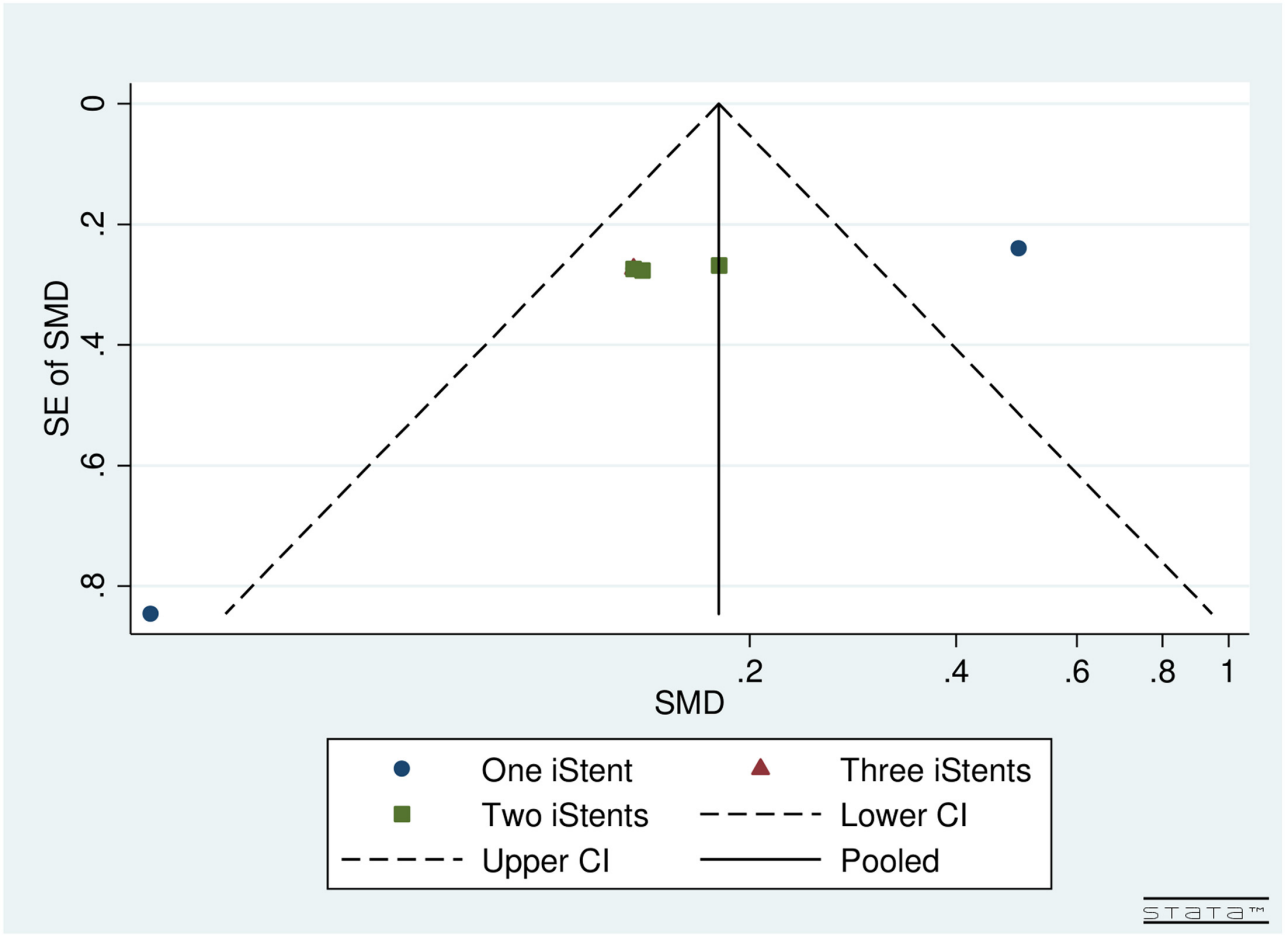
Funnel plot for studies examining pre- and post-operative number of medications by number of iStents inserted.

Visual inspection of funnel plots by follow-up and number of iStents implanted for both pre- and post-operative IOP (Figs 2 and 3) and topical glaucoma medications (Figs 4 and 5) did not reveal any asymmetry.

### Main outcomes

The main outcome of the study is to analyze and quantify the effect of iStent on both intraocular pressure and use of topical glaucoma medications.

### Effect on intraocular pressure

From Table 1, a 22% reduction in IOP (IOPR%) occurred at 18-months following an iStent implantation [32]. The weighted mean IOPR% after two iStents implantation at 6-months follow-up was computed to be 30%, where weights were determined by the respective study sample sizes [40,41]. A 41% reduction in IOP occurred at 6-months following the implantation of three iStents [40].

**Fig 6:**
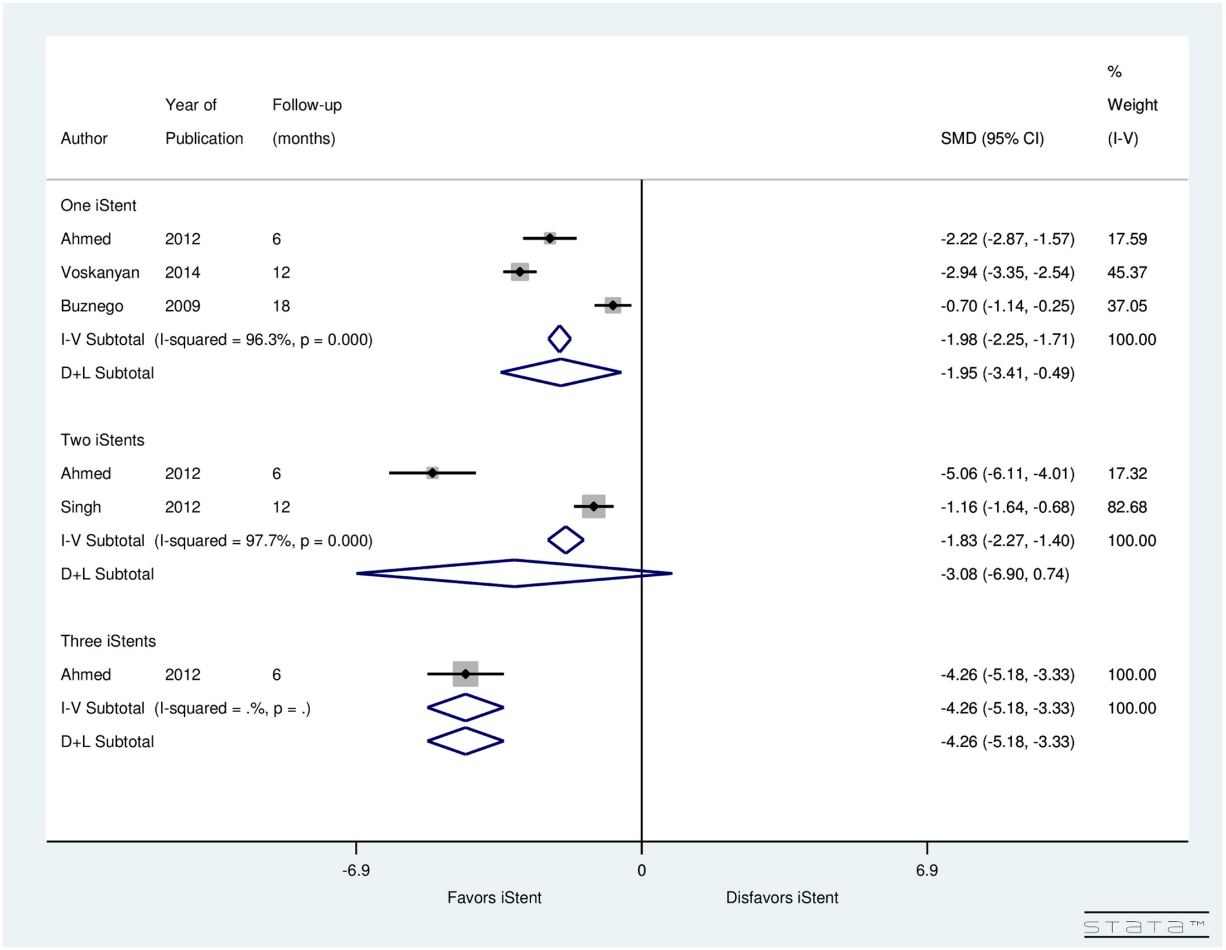
Forest plot for pre- and post-operative IOP by number of iStents inserted.

Fig 6 summarizes the results for the outcome measure, IOP by the number of iStents implanted. Five studies (248 subjects) looked into the impact on IOP due to one or more iStents implanted. All the studies showed a decrease in post-operative IOP. Heterogeneity between studies that investigated the impact of one iStent (I^2^ = 96.3%) and two iStents (I^2^ = 97.7%) was significantly high. In studies examining the impact of one iStent (SMD = -1.95, CI: [-3.41, -0.49]), two iStents (SMD = -3.08, CI: [-6.9, 0.74]), and three iStents (SMD = -4.26, CI: [-5.18, -3.33]), IOP reduced significantly after one iStent and three iStents implantation. However, a non-significant reduction in IOP occurred after implantation of two iStents. It should be noted that there were only two studies evaluating the impact of two iStents and one study evaluating the impact of three iStents. Thus, more studies are required to make concrete conclusions.

**Fig 7:**
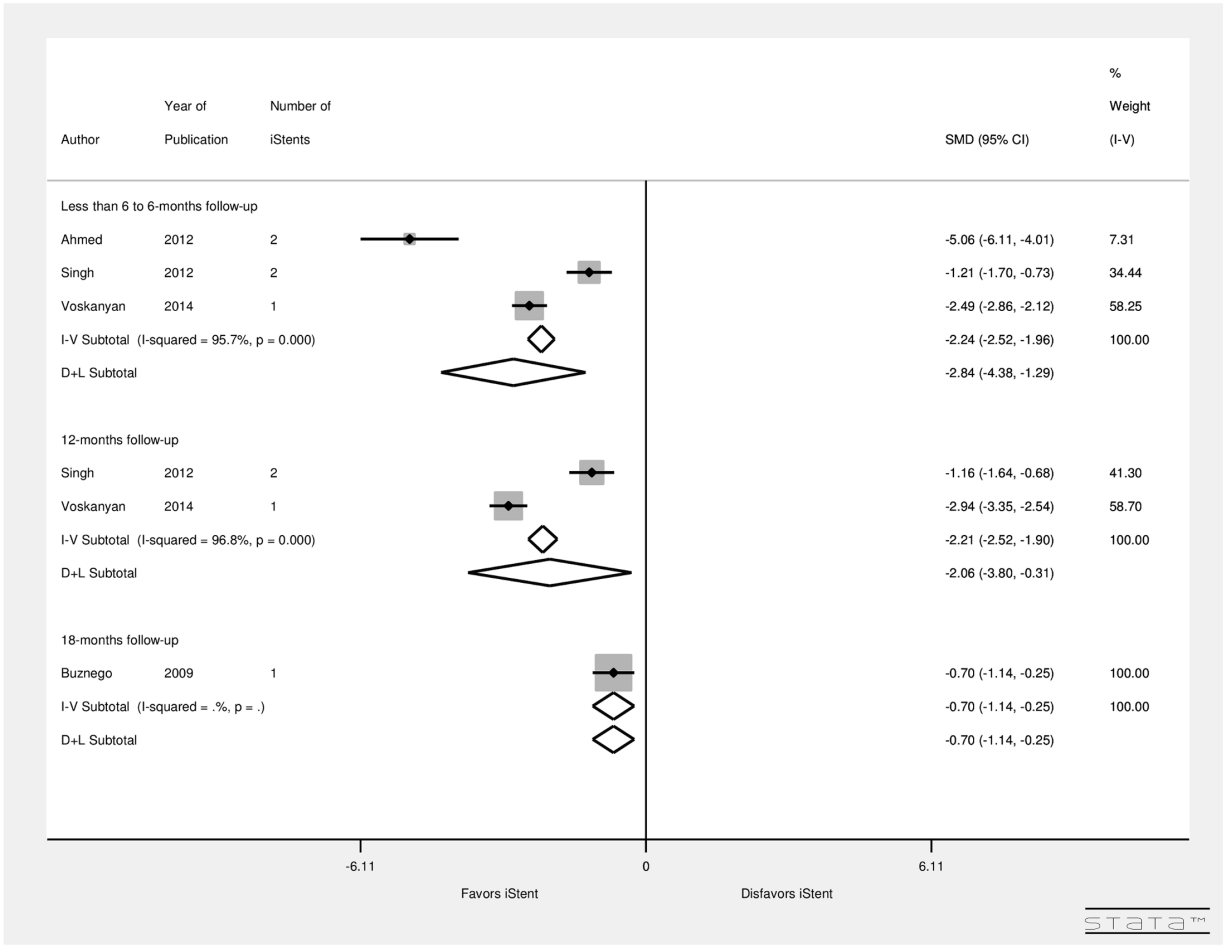
Forest plot for pre- and post-operative IOP by follow-up (months).

Fig 7 summarizes the results for the IOP outcome measure by the follow-up (months) for studies implanting one iStent, two iStents, and three iStents. There were 3 studies with a follow-up of less than 6-months to 6-months, 2 studies with 12-months follow-up, and a study with 18-months of follow-up. There was significant heterogeneity between studies examining less than 6-months to 6-months follow-up (I^2^ = 0.95.7%) and follow-up of 12-months (I^2^ = 96.8%). Fig 7 suggests that the maximum reduction in IOP occurred at less than 6 to 6-months (SMD = -2.84, CI: [-4.38, -1.29]). Additionally, significant reduction in IOP occurred at 12-months (SMD = -2.06, CI: [-3.8, -0.31]) after iStent implantation. Another study by Buznego [32] with 18-months follow-up showed a significant decrease in IOP levels (SMD = -0.70, CI: [-1.14, -0.25]). This suggests that after iStent implantation, over a period of time, effect on IOP decreases.

### Effect on topical glaucoma medication use

From Table 2, a mean reduction of 1.2 bottles per patient of topical glaucoma medications occurred at 18-months after an iStent implantation [32]. The weighted mean reduction in topical glaucoma medications after two iStents implantation at 6-months follow-up was computed to be 1.45 [40,41]. One bottle of medication per patient was reduced at 6-months following three iStents implantation [40].

**Fig 8:**
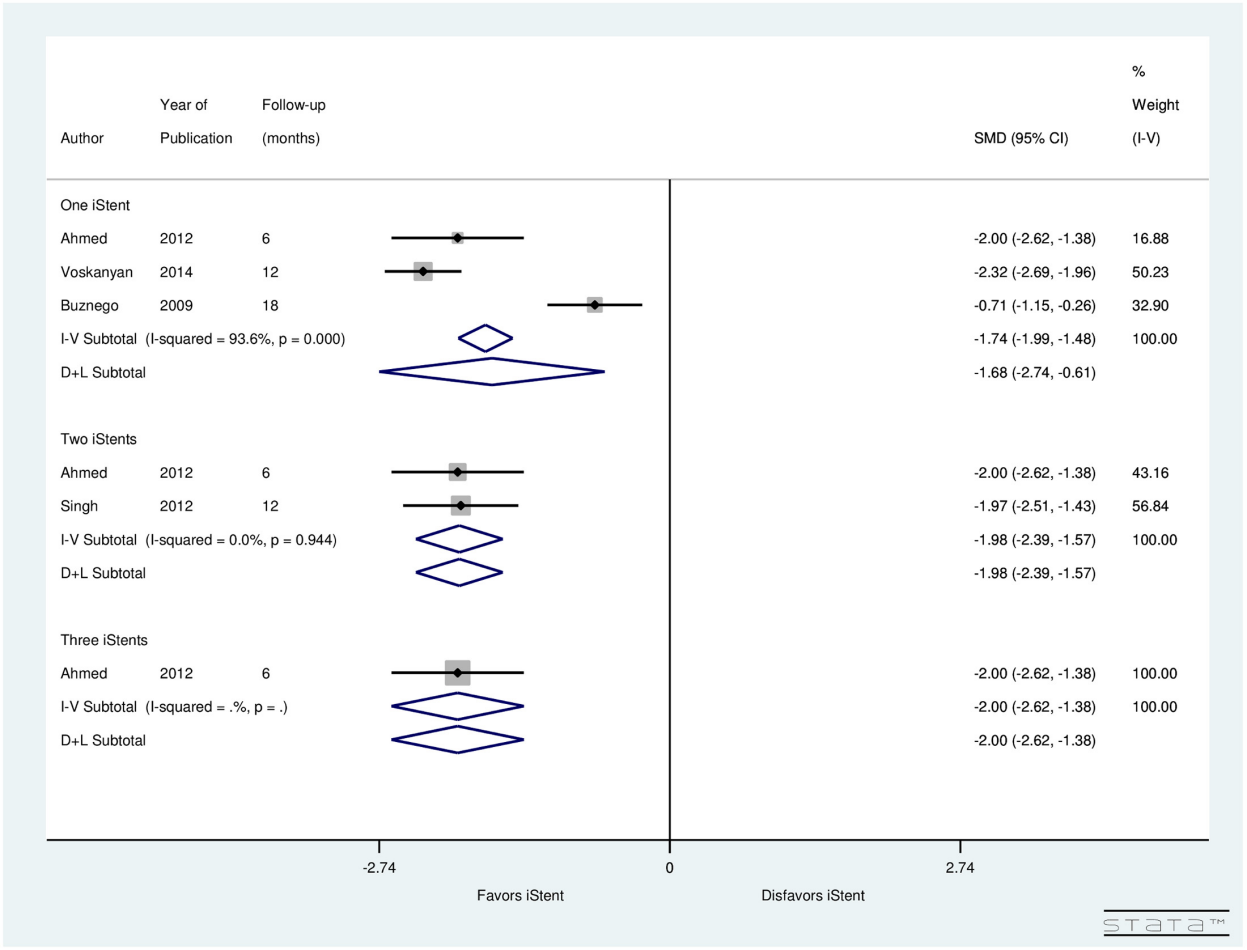
Forest plot for pre- and post-operative number of medications by number of iStents inserted.

Fig 8 illustrates a forest plot of SMD of pre- and post-operative number of medications with respect to number of iStents inserted. Studies examining the impact of one iStent showed a significant drop in the post-operative number of glaucoma medications (SMD = -1.68, CI: [-2.74, -0.61]) with a significant (*p* = 0.0) amount of between study heterogeneity (I^2^ = 93.6%). For studies investigating effect of two iStents had non-significant (*p* = 0.944) heterogeneity between studies (I^2^ = 0.0%) and random-effect computations indicated significant reduction in the number of glaucoma medications post-surgery (SMD = -1.98, CI:[-2.39, -1.57]). There was a single study assessing the impact of three iStents and it showed a significant reduction in the number of glaucoma medications (SMD = -2.00, CI:[-2.62, -1.38]) compared to one or two iStents. However, more studies examining the impact of two to three iStents are required to make solid conclusions.

**Fig 9:**
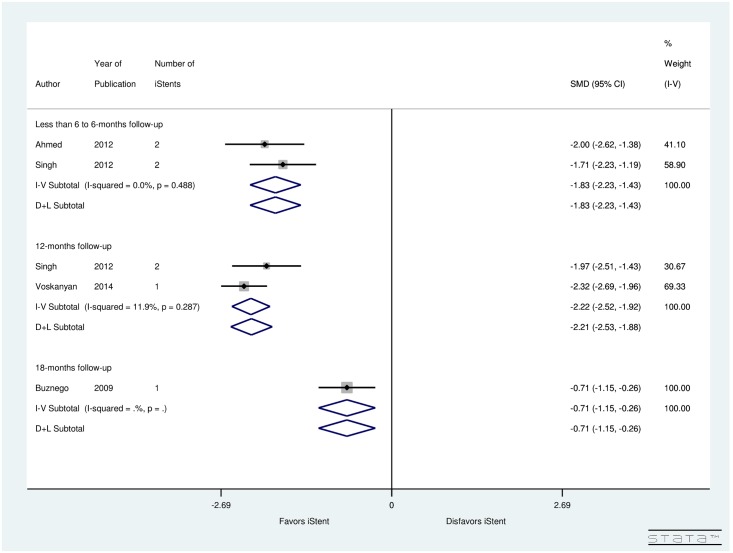
Forest plot for pre- and post-operative number of medications by follow-up (months).

Fig 9 shows a forest plot for SMD of pre- and post-operative number of medications by follow-up (months) for studies performing iStent in patients with glaucoma. There were 2 studies considering follow-up of 6 to less than 6-months, 2 studies inspecting 12-months follow-up, and a study with 18-months follow-up. Heterogeneity between studies examining follow-up of 6 to less than 6-months (I^2^ = 0.0%), and 12-months follow-up (I^2^ = 11.9%) was used to determine the random-effect or fixed-effect computations. Results showed significant reduction in post-operative number of medications in 6 to less than 6-months follow-up (SMD = -1.83, CI: [-2.23, -1.43]), 12-months follow-up (SMD = -2.21, CI: [-2.53, -1.88]), and even after 18-months of iStent implantation (SMD = -0.71, CI: [-1.15, -0.26]).

## Discussion

A systematic review of the literature by searching various bibliographic databases as well as the grey literature was performed to examine the effect of iStent implantation as a solo procedure without concurrent cataract extraction in patients with POAG. The principal outcome measures of change in the intraocular pressure (IOP) and number of topical medications were reported. Five studies (248 subjects) were included for qualitative and quantitative synthesis. Characteristics of the included studies such as study design, study population, study location, sample size, pre- and post-operative IOP and the number of medications, follow-up period, and number of iStent implanted were summarized.

Percentage reduction in IOP and mean reduction in topical glaucoma medications were also computed. A 22% IOP reduction (IOPR%) from baseline occurred at 18-months after one iStent implant, 30% at 6-months after two iStents implantations, and 41% at 6-months after implantation of three iStents. A mean reduction of 1.2 bottles per patient of topical glaucoma medications occurred at 18-months after an iStent implant, 1.45 bottles per patient at 6-months after two iStents implant, and a bottle of medication per patient was reduced at 6-months following three iStents implant. Meta-analysis results were positive and promising by demonstrating significant improvement in post-operative IOP and reduction in number of topical glaucoma medications after iStent surgery. A significant strength of this analysis stems from the fact that all the included studies had coherent results of improvement in IOP as well the number of topical glaucoma medications due to iStent surgery.

There was a considerable amount of heterogeneity among three studies examining one iStent and two studies investigating two iStent implantations. Random-effect computations showed significantly controlled and lowered IOP due to both one iStent and two iStents. Further, just one study [40] reported improvement in IOP after three iStents and showed that the improvement in IOP correlated with the number of iStents implanted. More studies are needed to fully understand this relationship.

Substantial heterogeneity between the studies was shown which could reflect different study populations, demographics, inclusion/exclusion criteria, study location, iStents implantation technique, surgeon’s experience, available facilities to perform iStent surgery, rates of complications, year the surgeries were performed, year the study was conducted as well as attention to placement related to collector channel identification. The results of this quantitative synthesis of the currently available literature suggest that more studies need to be reported to better understand the optimal role of the iStent in IOP management and topical glaucoma medication management.

The study limitations for meta-analyses such as this are necessary before inferences may be considered. First, it is necessary to consider the quality of the included studies. In this meta-analysis the Downs and Black checklist [38] was employed. This revealed a significant variation in quality scoring with high-, medium-, and poor-quality studies having been reported. Nevertheless, as only five studies were available for analysis, all were included, irrespective of their quality. This is a recognized, but necessary, limitation due to the few clinical studies currently available. Second, meta-analysis of observational studies is influenced by inherent biases in the included articles [43]. For example, a multitude of other factors such as level of education, ethnicity, income status, socioeconomic status, previous ocular and non-ocular surgeries, family history, other ocular and non-ocular diseases, pre-operative and post-operative medications, number of medications and comorbidities (e.g., high blood pressure, diabetes, stroke, heart conditions, etc.) could influence the estimates in the original studies. Potential bias related to industry sponsorship of a study also exists, as well as methods of patient selection. Variations in surgical technique may be a major factor as attention to placement near collector channels is known to increase the effect [39].

The results of this meta-analysis showed improvement in IOP and reduction in the number of topical glaucoma medications after iStent implantation. Although the data were limited, data suggest that the IOP decrease correlates with the number of iStents implanted. However the heterogeneity in the current literature suggests that additional research is warranted to best understand how to maximize the utility of iStent in the management of glaucoma patients. A notable lack of published research on rates of early and late post-operative complications suggests more research is also needed in this area. Similarly almost no current literature exists regarding the identification of factors that may be predictive of success such as previous ocular and non-ocular surgeries, family history, other ocular and non-ocular diseases, pre-operative and post-operative medications, number of medications and comorbidities as well as consistency in the stage of the disease.

In conclusion, this systematic review and meta-analysis has shown that the iStent as a solo procedure without concurrent cataract surgery does lower IOP and reduces the dependency on glaucoma medications. The effectiveness data in studies such as this are necessary to conduct cost-effectiveness (CE) and cost-utility (CU) analyses. As health care has evolved in the last decade into a much greater cost-effective model, data such as these would be useful for governmental agencies, clinicians, hospital administrators, payers, and policy-makers to better understand and define the position of newer technologies in existing treatment paradigms. The study also highlights the challenges that exist when devices are brought to market without large, prospective randomized trials with extended follow-up periods. It is only through a synthesis of data from smaller studies by early adopters that more widespread utilization and implementation by health care systems may be possible.

## Supporting Information

S1 PRISMA Checklist PRISMA 2009 Checklist. (DOC)

S1 File Search strategy for EMBASE and MEDLINE. (DOCX)

S2 File Level 1, 2, and 3 screening questions. (DOCX)
