# Supplementary material for: iStent as a Solo Procedure for Glaucoma Patients: A Systematic Review and Meta-Analysis
Source: PLoS One. 2015 May 27;10(5):e0128146. doi: 10.1371/journal.pone.0128146 (PMC4446040; doi:10.1371/journal.pone.0128146)
Supplement: S1 File — (DOCX) [file pone.0128146.s002.docx]

**S2: SEARCH STRATEGY for EMBASE and MEDLINE**

**EMBASE**

1     Intraocular hypertension/ or Open angle glaucoma/ (18819)
2     (Ocular hypertens* or Intraocular hypertens* or Intra-ocular hypertens* or Suspect glaucoma* or hydrophthalmos or Pigmentary Glaucoma* or Simple Glaucoma* or Open-Angle Glaucoma* or Glaucoma simplex or Open Angle Glaucoma* or Wide angle glaucoma*).mp. (22277)
3     1 or 2 (22277)
4     (istent* or micro invasive glaucoma* or micro-invasive glaucoma* or MIGS or trabecular micro-bypass* or trabecular micro bypass*).mp. (151)
5     4 and 3 (29)
6     limit 5 to (english language and yr="2000 -Current") (29)

**MEDLINE**

1     Ocular hypertension/ or exp Glaucoma, Open-Angle/ (13508)
2     (Ocular hypertens* or Intraocular hypertens* or Intra-ocular hypertens* or Suspect glaucoma* or hydrophthalmos or Pigmentary Glaucoma* or Simple Glaucoma* or Open-Angle Glaucoma* or Glaucoma simplex or Open Angle Glaucoma* or Wide angle glaucoma*).mp. (14370)
3     1 or 2 (17492)
4     (istent* or micro invasive glaucoma* or micro-invasive glaucoma* or MIGS or trabecular micro-bypass* or trabecular micro bypass*).mp. (99)
5     3 and 4 (19)
6     limit 5 to (english language and yr="2000 -Current") (19)
