# Supplementary material for: iStent as a Solo Procedure for Glaucoma Patients: A Systematic Review and Meta-Analysis
Source: PLoS One. 2015 May 27;10(5):e0128146. doi: 10.1371/journal.pone.0128146 (PMC4446040; doi:10.1371/journal.pone.0128146)
Supplement: S2 File — (DOCX) [file pone.0128146.s003.docx]

**S3: LEVEL 1, 2, AND 3 SCREENING QUESTIONS**

**Level 1 screening**

1. Does the study look at iStent or prostaglandin analogue or beta blockers or alpha agonist or carbonic anhydrase inhibitors in humans with glaucoma?
   1. Yes
   2. No
   3. Unclear
2. Is this a research study (not an editorial, opinion, case report or a review article)?
   1. Yes
   2. No
   3. Unclear
3. Does the study look at Primary open angle glaucoma or ocular hypertension or open angle glaucoma secondary to pseudoexfoliation.
   1. Yes
   2. No
   3. Unclear

**Level 2 Screening**

1. Does this study look at efficacy, side effects, or cost of iStent or prostaglandin analogue or beta blockers or alpha agonist or carbonic anhydrase inhibitors?
   1. Yes
   2. No
   3. Unclear
2. Is there an adequate follow-up to assess efficacy or side-effects or cost of iStent?
   1. Yes
   2. No
   3. Unclear
3. Does the study consider the sample size of 20 or more patients?
   1. Yes
   2. No
   3. Unclear
4. Is this a research study (not a pilot study or a survey considering percentage of people suffering from glaucoma)?
   1. Yes
   2. No
   3. Unclear
5. Have patients in this study not been on ALT or trabeculectomy?
   1. Yes
   2. No
   3. Unclear

**Level 3 Screening**

1. Are complications explicitly discussed and included in the paper?
2. Yes
3. No
4. Unclear
5. Are complications and outcomes (ie IOP, vision, compliance, quality of life) or cost or probability of occurrence of an outcome/complication explicitly discussed and included in the paper?
6. Yes
7. No
8. Unclear
